# Traumatic appendicitis: a case report and literature review

**DOI:** 10.1186/1749-7922-8-31

**Published:** 2013-08-09

**Authors:** Abdesslam Bouassria, Karim Ibn Majdoub, Issam Yazough, Abdelmalek Ousadden, Khalid Mazaz, Khalid Ait Taleb

**Affiliations:** 1 School of medicine and pharmacy of Fez, Sidi Mohammed Ben Abdellah University, BP: 1893; km2.200, route de sidi Hrazem, Fez 30000, Morocco; 2 Department of surgery, University Hospital Hassan II, BP: 1893; km2.200, route de sidi Hrazem, Fez 30000, Morocco

**Keywords:** Appendicitis, Abdominal trauma

## Abstract

Appendicitis and trauma may exist together, which causes an interesting debate whether trauma has led to appendicitis. We report a case of appendicitis after an abdominal trauma. Our patient developed acute appendicitis following a stab wound in the right iliac fossa. Surgical exploration confirmed the traumatic origin of appendicitis, appendectomy was performed and our patient made an excellent recovery. In non operative management of abdominal trauma, physical examinations and radiological explorations should be repeated in order to diagnose traumatic appendicitis.

## Introduction

Trauma and appendicitis are the commonest emergency conditions requiring surgery, especially in young adults. The pathological process in appendicitis generally starts with obstruction of the appendiceal lumen and may progress to peritonitis and development of intraabdominal abscess via appendiceal inflammation and perforation. An abdominal trauma may be responsible for damage of digestive tract or solid organs (liver or spleen). Occasionally, appendicitis and trauma exist together, which causes an interesting debate whether trauma has led to appendicitis. Actually, the role of abdominal trauma is still uncertain in the etiology of appendicitis. Blunt abdominal trauma or penetrating trauma like a stab wound may lead to an acute inflammatory response which is suggested to be the probable mechanism of traumatic appendicitis.

We report a case of appendicitis after an abdominal trauma (stab wound). To our knowledge, it is the first case of acute appendicitis after a stab wound reported in the literature.

## Case report

**Figure 1:**
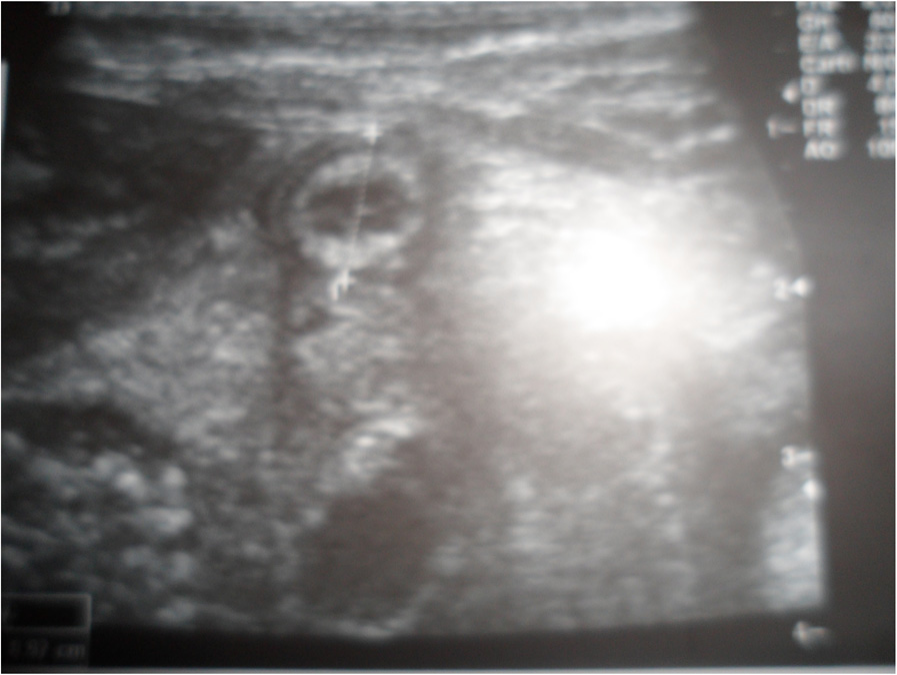
Abdominal ultra sonography of our patient showing appendicitis.

**Figure 2:**
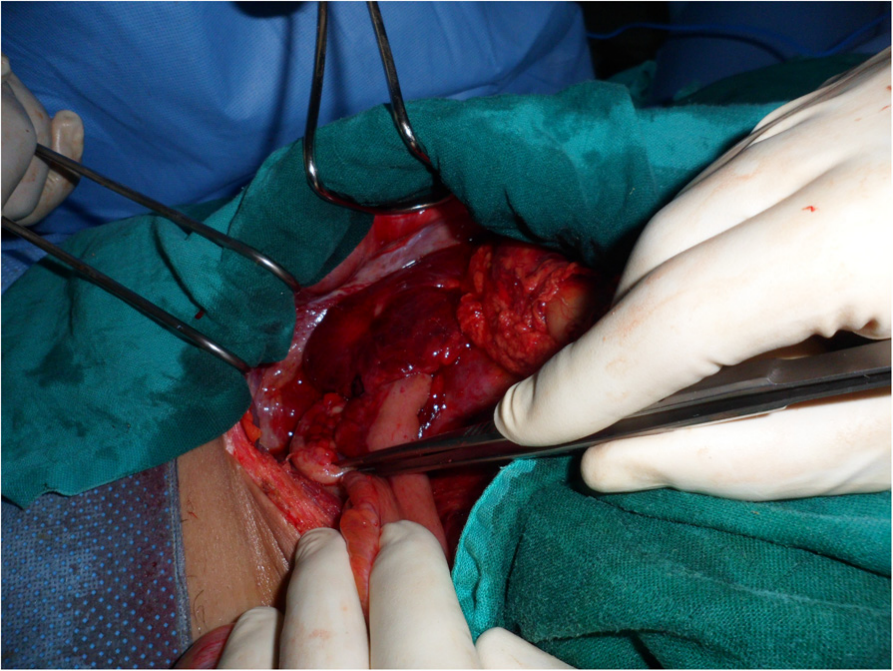
Intra operative photo showing the right para colic retroperitoneal hematoma and the appendicitis.

A 24 year-old man was admitted to the emergency department because of an abdominal injury following a stab wound which occurred on the same day. He said he was assaulted one hour before his admission by a stab wound in the right iliac fossa. His assailant used a sharp instrument (kitchen knife).The physical examination showed a conscious patient hemodynamically stable whose temperature was 37°C, whose pulse rate was 80 beats/min, whose respiratory rate of 20 breaths/min and whose blood pressure was 130/80 mmHg. Abdominal examination was normal out of mild tenderness at the abdominal wound which was located in the right iliac fossa. Laboratory investigations showed that the hemoglobin level was 12.8 g/dl, and the white blood cell count was 9800/mm3. Abdominal ultra sonography (US) was normal. So, a non operative management was decided. The penetrating abdominal wound (2 centimeters in length) was located in the right iliac fossa. It was disinfected and sutured. The day after his hospitalization, he had acute right iliac fossa pain. On examination, he was found to have a blood pressure of 120/80 mmHg, a pulse rate of 80 beats/min and a respiratory rate of 20 breaths/min; he was mildly pyrexial at 37.5°C. Abdominal examination revealed tenderness in the right iliac fossa. Laboratory investigations showed that the hemoglobin level was stable, but the white blood cell count was significant for a leukocyte count of 14,000/mm3 with 80% polymorphonuclear leukocytes. Then, abdominal US showed acute appendicitis (Figure 1). An emergency operation was performed. At laparotomy, a right paracolic retroperitoneal hematoma was detected. The patient had pelvic appendix in position. The appendix was hyperemic and edematous. Hematomas of the caecal wall and of the appendiceal wall were found (Figure 2). Appendectomy was performed. Histopathology confirmed diagnosis of acute appendicitis. Our patient made an excellent recovery, and he was discharged from the hospital in stable condition 2 days later.

This study was performed according to the declaration of Helsinki and approved by the Local Ethical Committee.

## Discussion

The acute appendicitis is the most common abdominal surgical emergency. It is an acute inflammation of the appendix related mostly with obstruction of the appendiceal lumen. This obstruction is usually caused by an inspissated stool, a mucus plug, or a foreign body [1]. Non-obstructive causes are also discussed such as bacterial invasion of the lymphoid tissue of the appendix [2]. Abdominal trauma was also mentioned as a possible etiologic factor in acute appendicitis. Interest in the association between appendicitis and blunt abdominal trauma may have begun with illusionist Harry Houdini’s untimely death in 1926: he is said to have died from a rupture appendix after a blow to the abdomen. During the 1930s, reports of blunt abdominal trauma and subsequent appendicitis began to appear [3] (Table 1). However, only few cases of minor BAT and TA have been reported in the literature, which may be attributed to the rarity or the difficulty to diagnose this relationship. Hennington and al. reported two cases of blunt abdominal trauma producing acute appendicitis. In both cases, blunt abdominal trauma has produced appendiceal edema with inflammation and hyperplasia of appendix lymphoid tissue, and then, obstruction of the appendiceal lumen, leading to acute appendicitis [4]. Ciftçi and al reported 5 cases of appendicitis occurring after abdominal trauma suggesting the same mechanism [2]. It is well known that intra-abdominal pressure increases in varying degrees in every blunt abdominal trauma case [5-7]. According to Ramsook [3], a sudden increase in intra abdominal pressure may lead to an increased intra ceacal pressure followed by a rapid distention of the appendix which may result in appendicitis.

**Table 1:** Review of the cases of traumatic appendicitis reported in the literature

**Year**	**Authors**	**Cause of traumatic appendicitis**	**Mechanism of traumatism**
1927	Richard J. Behan, Ann Surg. 1927 Feb 85(2):263–8.	14 cases	Bicycle Fall, Industrial accident
1940	G.K. Rhodes, California and western medicine, vol 53 n°4	7 cases	Abdominal trauma during scuffle, sports injury, industrial accident, car crash
1991	Hennington and al. Annales of surgery, 1991	2 cases	Industrial accident, Bicycle fall
1993 – 2002	B. Etensel and al. Emerg Med J 2005 22:874–877	5 cases	4 car crashes, 1 fall from a height of 10 meters
1996	A.O. C iftçi, and al.Eur J Pediatr Surg1996;6:350–3.	5 cases	Abdominal trauma
2002	Hager and al., Emerg Med J 2002 19:366–367	1 case	Fall from a ladder
2006	L. Pisoni and al. Ann Ital Chir. 2006 Sep Oct 77(5):441-2	1 case	Abdominal trauma
2010	Atalla MA and al.ANZ J Surg. 2010 Jul-Aug 80(7–8):572-3	1 case	Car Crash
2012	Paschos KA and al., Emerg Med Australas. 2012 Jun 24(3):343–6.	1 case	Blunt abdominal trauma
2013	Wani I. Post traumatic retrocecal appendicitis. OA Case reports 2013 May 01; 2 (4): 31	8 cases	Fall, Kicked in the abdomen, Bicycle fall

Serour and al have claimed that direct appendiceal injury is generally coexistent with other intra-abdominal organ injuries, and that the appendix is very rarely affected by direct trauma as it is very mobile and its dimensions very small [8]. As for our patient, hypothesis of appendicitis and abdominal trauma both existing together was easily dismissed because he was attacked by a sharp instrument. The stab wound in the right iliac fossa produced a penetrating abdominal wound. Then, the sharp instrument traumatized the meso colon and the meso appendix, causing the para colic retroperitoneal hematoma and hematomas of the caecal wall and the appendiceal wall. The result of these anatomic lesions was acute appendicitis due to the consequent luminal obstruction of the appendix.

## Conclusion

Appendicitis may follow abdominal trauma. Blunt abdominal trauma leading to appendicitis is rare, and occasionally, appendicitis and trauma exist together, which causes an interesting debate whether trauma has led to appendicitis. We report a case of abdominal trauma due to a sharp instrument which directly led to acute appendicitis. As the abdominal trauma was not a BAT, it was easy to relate the stab wound in the right iliac fossa to acute appendicitis. In non operative management of abdominal trauma, physical examinations, abdominal ultra sonography and/or abdominal computed tomography should be repeated for diagnosis of traumatic appendicitis in order to prevent potential complications of appendicitis.

## Consent

Written informed consent was obtained from the patient for publication of this case report and any accompanying images.

## Competing interests

All authors declare no competing interests.

## Authors’ contributions

AB and KIM participated in writing the case report and revising the draft, IY were involved in literature research and were major contributor in writing the manuscript. AO and KAT and KM participated in the follow up. All authors read and approved the final manuscript.

## Authors’ information

School of medicine and pharmacy of fez, Sidi Mohammed Ben Abdellah University department of surgery, university hospital Hassan II, BP: 1893; km2.200, route de sidi Hrazem; fez 30000, morocco.
